# Dr. Pratt’s Artificial Nipple

**Published:** 1846-01-01

**Authors:** 


*Dr. Pratt's artificial Nipple.*—We would invite the attention of our brethren to a little invention entitled as above, which is calculated to obviate most of the difficulties and sufferings so often incident to nursing women. We had an opportunity recently of witnessing its application by Dr. P. in a case of excoriation, and one of retracted nipple from its small size and over distension. In both cases it acted like a charm, affording instantaneous and complete relief. The instrument consists of a small metallic shield with a valve or opening, and a movable cap of gum elastic, which is an excellent substitute for the natural organ. It is really one of the most useful contrivances we have seen for some time, and we think Dr. P. is entitled to the thanks of the profession for devoting his ingenuity to this object. He has certainly laid mothers under great obligations to him. To use his own language, he says, "I am sure to be well received wherever I go, for I save the doctors from dodging, and the women from crying." He has also invented an instrument for drawing the breast, and a nursing bottle, which are preferable to any others we have seen. They may all be found at *A. I. Mathews* Drug Store.

